# Effectiveness of road safety prevention in schools

**DOI:** 10.3389/fpsyg.2022.1046403

**Published:** 2022-12-20

**Authors:** Silke von Beesten, André Bresges

**Affiliations:** Institute of Physics Education, University of Cologne, Cologne, Germany

**Keywords:** road safety, safety behavior, design-based research, youth risk, road accident prevention

## Abstract

The World Health Organization estimates that each year, 1.3 million people are killed and more than 50 million people worldwide are injured in road traffic accidents. According to a study conducted by the Allianz Center for Technology, more young people between the ages of 15 and 29 die in traffic accidents than as a result of illness, drugs, suicide, violence, or war events worldwide. That is about 400,000 per year, globally. Worldwide traffic accident prevention campaigns demonstrate the consequences of traffic accidents in an emotionalizing way in order to encourage drivers to adopt conscious safety behavior *via* adequate driving behavior. The consequences of traffic accidents are demonstrated by prevention campaigns often in an emotionalizing way to encourage drivers to adopt safety measurements through adequate driving behavior. Prior research suggests that the emotionalizing effect of the appeals must be accompanied by solution- as well as action-oriented and self-confidence-increasing measures, so that the instructive message is reinforced and does not lead to reactance. Thus, a strong need arose in the schools for a targeted training as the follow-up of emotional prevention campaigns. A suitable training for knowledge acquisition and knowledge transfer into everyday life was developed by means of the design-based research method. To create the targeted follow-up, various methods from cognitive behavioral therapy and common traffic safety programs were adapted. This publication is dedicated to a first explorative research approach in a non-standardized form of a social training. It approaches the question of negative emotional states immediately after a *Crash Kurs NRW* stage event, which is a prevention program in Germany that targets upper middle school and high school courses and originates from North Rhine-Westphalia. Changes in social behavior and development of participants' own norms, values, and attitudes were observed and documented and are discussed and presented in this article. The result of the survey confirmed prior research and showed visible effects of reactance after the Crash Curs NRW campaign. It was found that a structured follow-up training is suitable to gain reactive behavior from the stage event. Knowledge deficits about the cause and outcome of accidents were successfully addressed in the follow-up. This may have influence the reactance behavior and could be a key factor for successful prevention campaigns. Further publications will observe the connection between knowledge and reactance in subsequent iterative passes of modified follow-ups for the Crash Course NRW Campaign.

## Introduction

Mobility stands for economic growth and the participation of each individual in social life. It is particularly important for young, adolescent people in the phase of detachment from the parental home and networking with the peer group (Bastian, 2010, p. 24).

However, there is evidence that the mobility also has negative consequences: in total, 400,000 young people between the ages of 15 and 29 die each year in road traffic accidents worldwide (Allianz, 2014).

Consequently, traffic accident prevention and safety work enjoy a high level of social attention.

Prevention strategies are divided into universal, population-based, and area-based vs. target group-specific measures, which are declared, for example, by their sociodemographic characteristics or by their risk status (Thapa-Görder and Voigt-Radloff, 2010, p. 19). As a result of the causal relationship between vehicle and infrastructure-related factors and traffic management, the number of accidental deaths is much higher in low-income countries, for example (Faus et al., 2021a). In addition, especially in emerging countries, parameters such as the dependence of funding on government sources, fragmentation of decision-making processes in multidisciplinary areas, legal frameworks for road safety, public awareness, local needs, and institutional capacity for road accident prevention work are major determinants of advancing influential safety work (Eusofe and Evdorides, 2017).

Young drivers in the adult life phase are included in the high-risk group. The reasons for this classification are the detachment from the parental home, the search for identity, formative and groundbreaking changes in social relationships, orientation to the social peer group, and finding one's own social status. Testing one's own limits is typical and immanent in the adolescent phase of development.

For most young adults, this critical development phase includes the acquisition of a driver's license and, associated with this, greater access to individual mobility (Raithel, 2011, p. 9). Further causes for a high level of involvement in accidents lie in the additional criteria of “novice risk” and “youth risk” (Jugendlichkeitsrisiko, Landesverkehrswacht MV, www.verkehrswacht-mv.de, 18.06.2022). Novice drivers lack experience in dealing with motor vehicles and traffic situations. Risky behavior in dangerous situations is more likely to be accepted. As unsafe driving style develops, the car becomes a symbol of freedom (Bastian, 2010, p. 47).

This risk acceptance influences the assessment of the dangerousness and leads *via* incentives (cost-benefit) toward dangerous behavior (Seifert, 2007, p. 1), further in sum with the “beginner's risk” to an increased hazard potential in road traffic (Jugendlichkeitsrisiko, Landesverkehrswacht MV, www.verkehrswacht-mv.de, (18.06.2022).

Young people also use the traffic area as a sports and communication space and as a meeting place for group activities. Important social functions are controlled in the social reference group (“peer group”), where acceptance and recognition are also and especially found for risky behaviors. Consequently, age-typical dangerous behavior is to be seen as a main risk variable (Limbourg, 2013).

The purpose and type of traffic participation define different hazard potentials (Limbourg, 2013). Speeding violations, also combined with a lack of distance to the vehicle ahead, driving under the influence of alcohol or drugs, overtaking errors, red light violations and distractions, for example, by cell phones, lead the statistics for the causes of accidents among young drivers. Often, not wearing a seat belt is added as an injury-increasing criterion (www.destatis.de).

Therefore, road safety work must target this problem in a methodologically selective manner. These high-risk strategies are target group-oriented, but usually of higher cost-effectiveness than population-based strategies (Thapa-Görder and Voigt-Radloff, 2010, p. 19). In addition to penalty-based incentive and punishment systems, the focus is on the educational procedures (Thapa-Görder and Voigt-Radloff, 2010, p. 19). These are implemented and put into practice in educational institutions.

## Theoretical framework

### Prevention in the area of tension between practice and science

Cooperations among the police, local authorities, traffic guards, associations, and other institutions make it possible to communicate traffic accident prevention in a sustainable manner.

The police identify current traffic accident phenomena and hot spots, initiate prevention projects and, if necessary, participate in them (Verkehrssicherheitsarbeit der Polizei, https://www.recht.nrw.de, 06.09.2022). Police is present every day in the context of education and upbringing with its prevention work at educational institutions; indeed, it moves continuously and acts pedagogically in the areas of tension that arise from the encounter of pedagogical activities and core police work (Kepura, 2021, p. 278). Schools and the police encourage young people to become independent-minded, responsible personalities in the area of conflict between freedom and rules.

The school's educational mission should also convey the meaning of norms and reflect on them critically. Democracy-related educational goals such as autonomy, maturity, and the ability to participate and reflect are thus captured (Dewey, 2016).

Researching prevention work across systems (here: pedagogy and police) opens up greater perspectives of knowledge than the theoretical framework of only one scientific discipline, because the knowledge generated then does not remain entrenched in the inherent logic of the respective system (Steffen, 2012, p. 40).

In addition, studies have shown that communication campaigns, related to road safety messages, can be significantly increased in the effectiveness when accompanied by traffic education activities (Faus et al., 2021b).

### Behavior change through fear appeals

Road safety campaigns aim to change the behavior of their participants. Toward that goal, some campaigns aim at positive behavioral change in favor of health regularly act with the conception: “Create fear!”.

The cognitive dissonance of the perhaps deadly, but fast car ride with fun factor, must be resolved in favor of future orientation for the individual.

Newly propagated behavior patterns are to be strengthened, and the old ones renounced (Bonfadelli and Friemel, 2010, p. 56).

If the fear appeal is too strong, it can result in reactance (Dahlgren, 2021, p. 153, Dillard and Shen, 2005, p. 144). When people are exposed to content they have not asked for, they may see that as a threat to their received freedom. They may restore their freedom by becoming angry and counterarguing, or in some cases by choosing actions consistent with their prior attitudes since forced exposure can “subjectively decrease the attractiveness of the imposed alternative and increase the attractiveness of the denied option” (Dahlgren, 2021, p. 153).

The recipient denies the threat but does not change his behavior as desired, or even behaves in the opposite direction. This is often referred to as the “*boomerang effect“* in social psychology (Rossmann and Hastall, 2019, p. 435).

As early as the 1960's and 1970's, theories were developed that dealt with behavioral change through punishment or fear (Rogers, 1975, p. 93–114). These ”*fear appeal theories“* could not be empirically proven in their effectiveness (Ehlert, 2002).

According to the recent research, fear appeals are only effective if coping skills are promoted at the same time and action goals, action outcomes, and self-efficacy expectations are strengthened (Koehler et al., 2022, p. 3).

In addition, there is the finding that the effect of fear appeals is influenced by the individual differences in self-esteem (Leventhal and Hirschmann, 1982, p. 183–226).

The form and content of the formulated message are thus substantial, and the personal possibilities of the recipient determine how they are received. This determines the transport of content (Witte and Allen, 2020, p. 591–615).

First, the confrontation with the risk takes place, where the recipient first evaluates the risk. Positive and risk-minimizing behaviors are also presented. If the risk is assessed as low, no further processing takes place, and the new behaviors are not considered further. If the risk is rated as high, two things can happen: If one's own self-efficacy is perceived as sufficient to counter the risk with the help of the presented new behaviors, then the new behaviors are adopted. If the risk is rated high, but one's own self-efficacy is rated too low, and the new behaviors are rated as not suitable, not feasible, or unrealistic, then the new behaviors would not adopted, but rather the risk would be denied.

The US-American Psychologist Martin Seligman described this state as that of “learned helplessness,” which provides a suitable breeding ground for the development of, for example, depression and anxiety and thus in turn reduces self-worth (Seligman and Petermann, 2016).

Hackenfort et al. (2015, p. 215) therefore argue that a targeted follow-up is indispensable to implement traffic safety relevant behavior. This means that messages in themselves must be logical, comprehensible, and practicably applicable. Their meaning must be understood, and then, the new knowledge is readily applied.

This finding is supported by knowledge from health research studies on AIDS campaigns in the USA, which found that mass media campaigns as a prevention strategy can be successful in reducing AIDS under certain conditions and with careful planning and good execution, preferably with a focus on positive messages (Zatonski and Herbec, 2016).

In addition to these aforementioned criteria that influence readiness for behavior change, there is another important factor: recall of previously received safety messages at the right time in a traffic setting. A study of traffic safety behavior in the Dominican Republic focused on the human factor of remembering traffic safety campaigns with the result that only male professional drivers who had a driver's license and drove regularly were most likely to remember safety-related campaign messages (Faus et al., 2021b).

Thus, when these criteria are considered, the opportunities for effective road safety management increase.

### Potential stress reactions

The presented knowledge about the mode of action of fear appeals and the fact that these effects anchor themselves in the thoughts and emotional world of the addressee leads to the implied question of what consequences these triggered emotions have through the use of confronting media.

The risk of psychological overload through the presentation of the emotionally overstraining elements of the stage event and the psychological disturbance patterns that could be triggered by this seems to be given.

Overloads according to ICD 10 are in particular:

- F43.0 Acute stress reaction – without disease value, but with the risk of decompensation and an associated malaise.
- F43.2 Adjustment disorder–with disease value.
- F43.1 Post-traumatic stress disorder–with illness value (www.icd-code.de/icd/code/F00-F99.html).

This requires the preparation of the schools, in which students in risk of a post-traumatic stress disorder are detected and excluded from the stage event.

Finally, a well-prepared follow-up is required in which the contents are finalized by specific options for action in order to strengthen the learned messages and to prevent the scientifically known negative consequences of the so-called fear appeals.

### Research questions

Causally, the findings so far in this paper lead to the following key research questions:

*Q1: Which didactic methods are suitable to extract and* strengthen *the safety messages of the stage event?*
*Q2: Which didactic methods are suitable to influence dangerous behavior in an empathy-expanding way?*
*Q3: How must a follow-up module be designed in terms of content in order to be able to attach important road safety messages in the long term?*
*Q4: Was reactive behavior generated by the Crash Course NRW stage event and could it be minimized through follow-up modules?*

## Materials and methods

### Overview of the entire research

The design-based research method allows a systematic approach to these complex problems (Plattner et al., 2016).

We look at the problem through the user's lens.

In six different phases, the logic of the successive course of projects is measured against the milestones in order to drop or pass process steps if necessary.

Through iteration, the sequence of process steps through loops to previous phases. In this process, openness to results and a culture of mistakes are implemented: Because every failure, if recognized early, is a gain for the progress of the innovation process (Gerling and Gerling, 2018).

### Initial design

Based on the complexity of a traffic accident prevention campaign in all schools of an entire federal state for students between 16 and 24 years of age, it seemed obvious to use the research design of the design-based research method. This design promised sufficient systematic structures to be able to capture the wealth of needs and still focus on the necessary goals and promote their further development. In this study, we conducted research accompanying a Crash Course prevention program specifically addressing young drivers and passengers in their peer group.

The stage event took place 1 week before the follow-up.

In the stage event, the police presents the course of the rescue chain with actors and affected people as well as the events at the scene of the accident in an informative way.

With emotional biographical reports and vivid pictures, police and fire department officers, paramedics, emergency doctors, emergency chaplains, or even relatives of accident victims report on the causes and consequences.

Personal experiences such as the scene of an accident, first aid, or the news of a death are included.

Together with the use of confronting media, strong emotions are triggered in the participants.

As a state campaign by the police of North Rhine-Westphalia in cooperation with educational institutions, the Crash Course NRW prevention program specifically targets young drivers and passengers in their peer group, aiming to convey the following statements:

- Traffic accidents have a cause and do not just happen.
- Traffic accidents are avoidable.
- Deliberately disregarding traffic rules is a main source of traffic accidents.
- Important traffic rules are as follows: control your speed, buckle up, do not drink and drive, and do not distract the driver (or let other distract you as a driver).

The Crash Course NRW state campaign is designed as a program with three phases, in which schools and the police work together.

**Figure 1:**
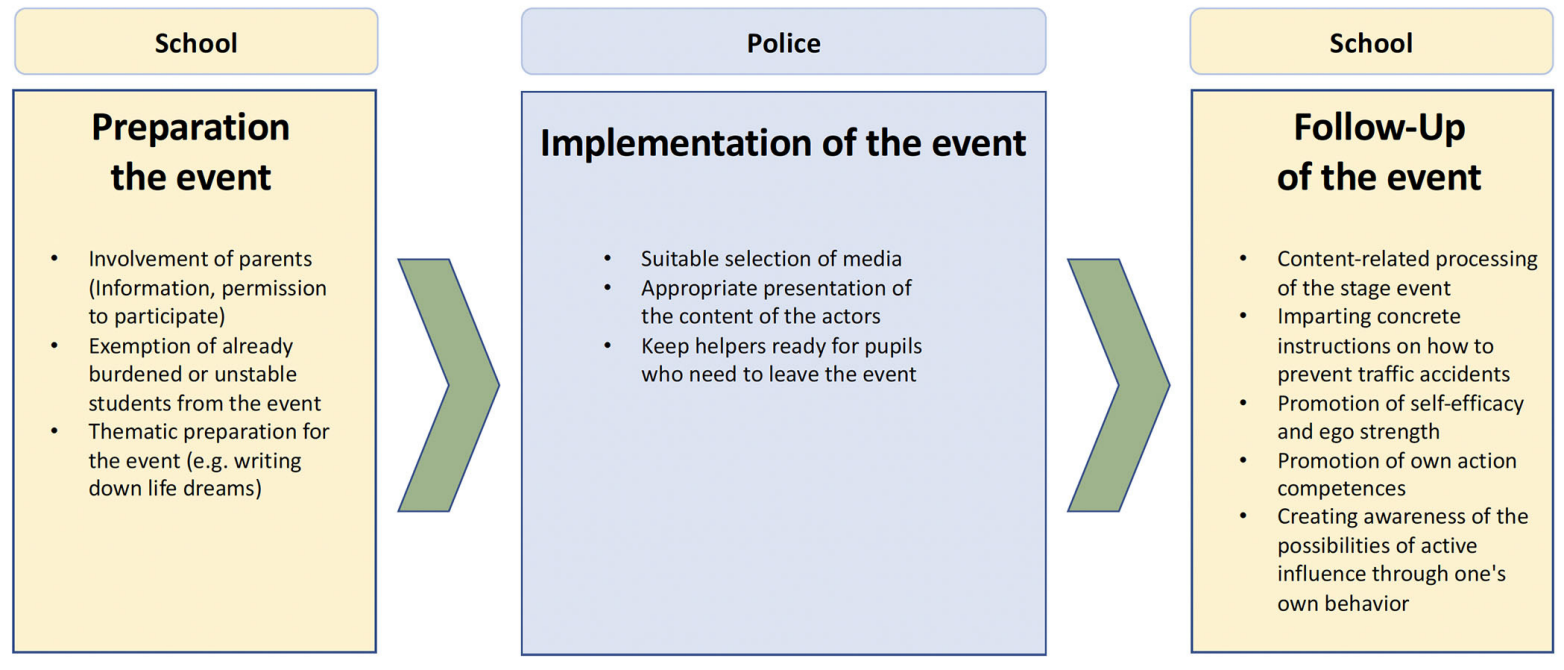
Overview of the phases of action of the Crash Kurs NRW.

The Figure 1 shows the three different phases with possible interventions to support proportionality and avert danger.

### Methods

The aim of this exploratory study was to uncover how people construct their reality, how functional or dysfunctional this constructed reality is, and how it needs to be restructured in order to achieve traffic safety-relevant behavior. Through an explorative examination of the risks of road traffic in conjunction with the information obtained through one's own possible actions, derivations for a changed future-oriented behavior and thus also stimuli for the cognitive restructuring of one's own norms, values, and attitudes can be created (Kleebein et al., 2010, p. 19).

In a secondary school class in the *Rheinisch-Bergisch* district, with *n* = 86 students, the explorative method is applied in the form of social training in order to gain insight into initially unstructured situations.

Returning to the research questions, the categories in which they were applied and the methods used to inquire about them are outlined below:

Q1 and Q2: Evaluating didactic methods of self-reflection, cognitive restructuring, change of perspective, and emotional reassessment by field-testing a targeted follow-up training, including the exercises “*Risk Assessment,” “Alcohol Impairment Goggles Memory,”* and the role play “*The Last Two Minute.”*
Q3: The main causes of accidents are discussed within a focus group.
  This moderated and focused discussion enables the participants to debate on behavioral measures that, on the contrary, lead to traffic safety behavior instead of risky behavior.
Q4:Application of special questions in both a pretest and post-test, which give indications of reactance. In addition, during the event, we observed whether there were any corresponding derogatory remarks or dismissive behavior, e.g., increased cell phone use or conspicuous staring out the window.
  The research survey was conducted exclusively with an online survey instrument.

The implementation of the intervention measure took place in the respective class in a familiar environment. In favor of the largest possible usable activity area, a circle of chairs with a centrally located open area was organized.

#### The risk assessment

By creating a risk assessment (see Appendix risk assessment) along a marked line between the poles ”Dangerous“ to ”Harmless,“ the participants themselves define the subjective matter of danger and the dangerousness of different traffic situations and discuss them.

Participants then reviewed their assessment in class as well as in facilitated discourse and reassessed if necessary.

Individual maps or hazards have been picked up and repositioned again and again.

The pros and cons of hazard assessment as well as the presentation of the current legal situation regarding these maps formed the core of the accompanying moderation.

Guided discovery as a technique from cognitive behavioral therapy serves the cognitive restructuring. One's own view of things and the perspective taken is then also reconsidered with the help of a Socratic dialog. Uncovering and moderated entanglement in contradictions make it clear that misbehavior in road traffic is without advantage and only seemingly logical. By creating confusion, distorted beliefs can be reframed and dysfunctional biases can be restructured into realistic assessments (Revenstorf and Burkhard, 2015, p. 256).

The view from the meta-level leads to the realization that the previous way of thinking is only one possibility among many and that other perspectives are just as realistic (Beck, 2013, p. 223 ff.) (Table 1).

**Table 1:** Overview of disputation techniques from cognitive behavioral therapy according to Wills, 2014.

Logical disputation style (reveals contradictions within thinking)	Driving fast is fun and gives a feeling of freedom, but what are the consequences and are they also a guarantee of freedom?
Empirical disputation style (points out differences between reality and thought)	Is it just driving fast, which is fun, or can I do something else with the same effect but more safety?
Hedonistic disputation style (explains advantages and disadvantages of certain thought patterns)	Is speeding a suitable method of maintaining freedom in the long term? Does it also have disadvantages?

**Figure 2:**
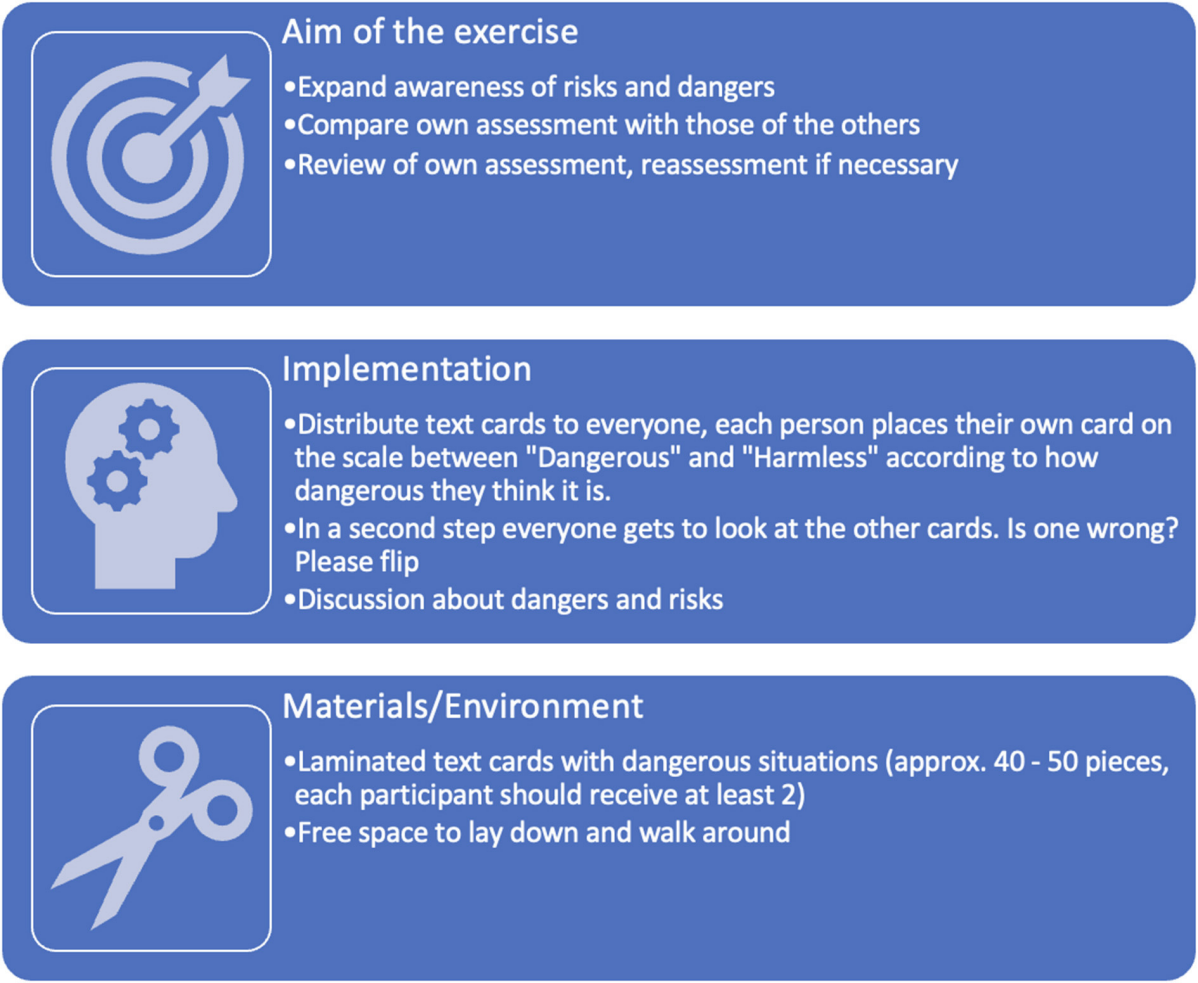
Overview of the “Risk Assessment” exercise.

As a result, a process of cognitive restructuring can be initiated and sustainably anchored in these exercises. The joint discovery in the peer group, guided by the facilitator, makes this possible (Figure 2).

#### The ”fatal vision alcohol goggles“–memory

**Figure 3:**
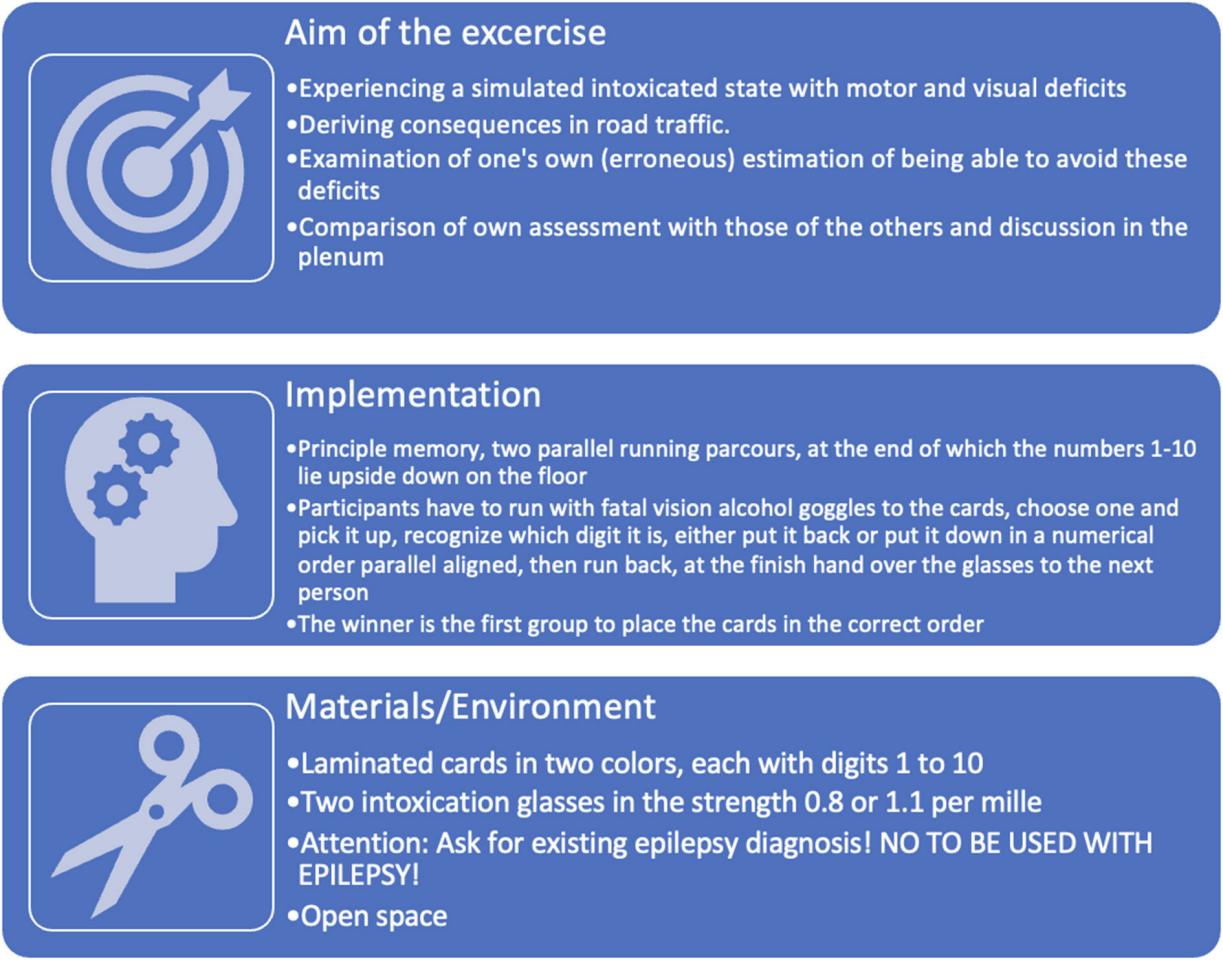
Overview of the “Fatal vision alcohol goggles” exercise.

Police, Deutsche Verkehrswacht (German road safety organization), ADAC (General German Automobile Club), schools, and other institutions use *fatal vision alcohol goggles* at campaign days and driver safety training courses to warn against alcohol consumption. The fatal vision alcohol goggles can simulate different levels of intoxication, produce limited all-round vision, double vision, misjudgments for proximity and distances, confusion, tunnel vision, delayed reaction time, and the feeling of insecurity (see https://www.lwl.org/ks-download/downloads/TakeCare/Toolbox/additional_exercises/Rauschbrille_Drunk%20Buster_Germany.pdf, 08.09.2022). The simulation refers to the representation of some intoxication effects as they can be found with increasing blood alcohol concentration (BAC) in the literature (Just, 2020, p. 464). Figure 3 shows the own experiences with the fatal vision alcohol goggles.

The effects observed in the exercise are used for a targeted evaluation and are used to check dysfunctional assumptions. In the exploration, an exchange of experiences about the effects of alcohol takes place, and legal basics and consequences are explained.

#### The role play ”The last two minute”

Participants simulate a driving situation in which they can actively influence events through their own actions.

The typical causes of serious injuries in road traffic had been communicated to the participants through the previous exercises and were therefore familiar to them.

However, knowing something does not necessarily mean protecting oneself from it. Initiating a specific action requires more than just “*the desire to do it* and the *knowledge of how to do it”* (Weinert et al., 1987, p. 3), according to Heckhausen and Gollwitzer's “Rubicon model.” Furthermore, it is necessary to be able to access behavior that has already been actively performed, which can then also be retrieved during stress.

The recall of an already known behavior, an already thought-out course of action, can also be possible in stress according to the theory of “*embodied cognition*” by Margaret Wilson. According to this theory, body, mind, and environment influence each other in thinking, feeling, and acting. Our thoughts trigger embodied reactions and *vice versa* (Wilson, 2002, p. 625). That means, any perception, both positive and negative, is stored as body memory with the corresponding physical attitude at that experienced moment. In the present role play, that would be the assumption, the experience at the time of the driving simulation, including the associated body posture and behavior, would be completely remembered in a later re-experience and could be reproduced as an automatism (Wilson, 2002, p. 634).

In this role play, students are asked to put themselves in the situation of being in a vehicle 2 min before a fatal crash. The social situation in the vehicle is presented to all the students by the following introduction:

*Through a conversation with the parents of one of the people involved in the accident, we know that the couple in the front seats were arguing when they left the parents' house. Jan and Marc are best friends, and Marc would never publicly criticize Jan, even if he made a driving mistake. Steffi is in a particularly awkward position socially: she has only been dating Marc for 2 weeks and is being taken out for the first time by the group in the evening. If she speaks up critically, she risks making a bad impression*.

Exercise assumption: all occupants of the car were killed in a collision at night with a roadside tree. (Source: https://crashkurs-nrw.uni-koeln.de/handeln-ueben, 08.09.2022). Role-playing builds up routines, behavioral sequences, and reactions. By trying out new behaviors, basic assumptions are changed, which in turn leads to new skills. Participants detect their own limits and weak points in critical situations and can work out solution scenarios (Beck, 2013, p. 257).

At the beginning, the role play offers a perspective transfer from the outside role to the influential driver and passenger role and puts the participants in the position of actively leading a car ride into disaster.

The deliberate bringing about of a catastrophe with a subsequent analysis of the risk factors should reveal the protection opportunities to the students that could have contributed to avoidance of the accident (Beck, 2013, p. 226–35).

*Example: loud music distracted the driver. Derived protective behaviors: Turn down music in vehicle or turn it off altogether*.

We use participant observation to explore whether this type of pedagogical role-playing is suitable for identifying risky actions in drivers. Furthermore, we test whether the co-drivers can recall and apply rehearsed actions to avert risky behavior under the simulated realistic conditions.

**Figure 4:**
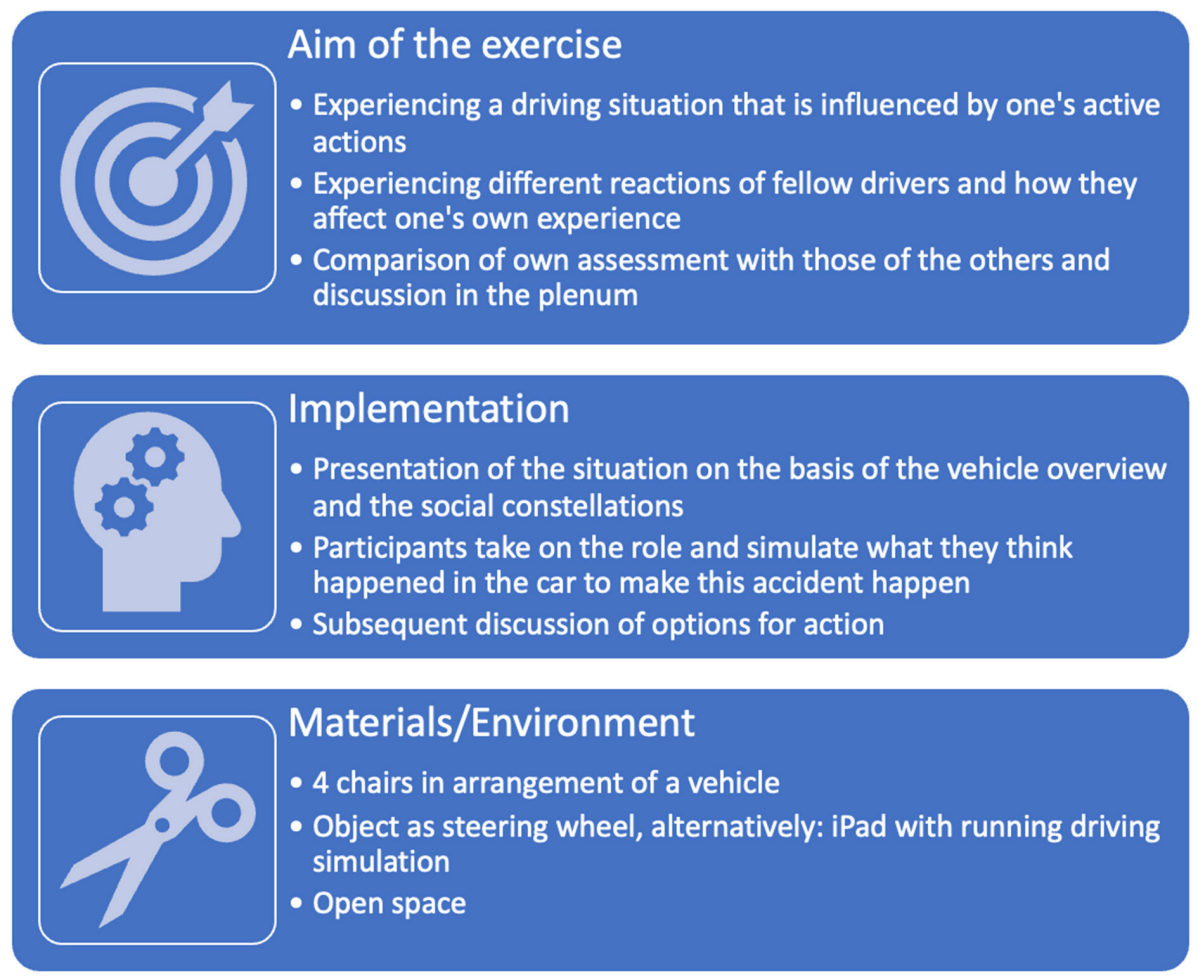
Overview of the exercise “The last two minute.”

Intended Goal: Pedagogical role plays should enable recognition of dysfunctional actions and lead to more safety- and risk-conscious behavior by practicing modified functional actions. In Figure 4 the role play is presented.

In addition to the “Driving Situation” exercise, the following action strategies can be discussed:

- Where does a drunk passenger sit in the car? The result of the discussion should be: if possible, at the back on the right–furthest away from the driver under the supervision of others–without the possibility of directly influencing the driver and negatively affecting his or her driving actions.
- How can the passenger actively protect the driver? For example place arm on the backrest and separate the driver from the rear, observe what is happening in the car and intervene if necessary.
- Does loud music influence driving? Loud music influences concentration.
- What can I do as a passenger if I really want to get out of the vehicle? For example, feign nausea and vomiting.
- How do I deal with the situation if the driver is obviously unfit to drive before starting the journey? For example, take away the keys, call the police, do not get in the car myself.
- What options do I have for getting home safely if the scheduled driver drops out? For example, cab, calling parents or other friends, and public transportation.

## Results

### Results of quantitative methods

#### Results preliminary research

In order to capture the thematic needs of participants coming to the follow-up after a Crash Course stage event, an online survey of 86 participants was conducted 1 week after the stage event. The purpose was to clarify, prior to the intervention, which content-related questions remained unanswered among the target group after the stage event. Furthermore, the studies by Hackenfort suggest that reactance may already be present in some participants following the confronting stage event. The extent of this reactance should to be quantified with this survey. The survey conducted using the LimeSurvey® tool yielded the following results: 2020, secondary school grade 9, follow-up Crash Course NRW, *n* = 86 (complete responses: 71, dropouts: 6, did not participate in electronic survey: 9). Out of this 71:

- Twenty-two participants (31%) were taking part in the “Accompanied driving from 17” program at the time of the survey.
- Fifty-two participants (73%) felt sorry for the actors (SQ001).
- Sixty-seven participants (94%) wanted to prevent something like this from happening to them too (SQ002).
- Fourteen participants (20%) did not know at all/not at all that accidents can have these consequences (SQ003).
- One participant (1,5%) was “fully afraid”/5 participants (7,5%) were “rather afraid” to drive a car themselves (SQ004).
- Fifty-five participants (77%) found it important to talk about traffic accidents (SQ009).
- Forty-one participants (58%) already knew all this (SQ005).
- Fourteen participants (20%) were annoyed (SQ006).
- Ten participants (14%) would have preferred to go outside (SQ007).
- Forty-one participants (58%) will recommend the event to others (SQ008).
- Forty-two participants (60%) wanted causes of accidents to be addressed in follow-up (SQ001).
- Fifty-two participants (73%) wished that it was addressed how an accident can be prevented (SQ002).
- Fifty-one participants (71%) wished that it was addressed which situations are dangerous in road traffic (SQ003).
- Thirty-four participants (48%) wanted to know who takes care of the victims after a traffic accident (SQ004).
- Forty-six participants (65%) wanted to know more about legal conditions (SQ005).
- Forty participants (56%) wanted to learn more about the influence of health and traffic risk (SQ007).
- Forty-four participants (62%) wanted to know exactly what alcohol, drugs and medication do (SQ006).
- Two participants (10 %) indicated that they had no interest in follow-up at all (SQ008).

#### Results of the post-test

After the follow-up training, a quantitative survey was again conducted using the survey tool (2020, secondary school class 9, follow-up Crash Course NRW, *n* = 86 (fully answered: 57, dropouts: 15, did not participate in the electronic survey: 14). Out of this 57:

- Thirty-four participants (59%) consider distraction to be a dangerous risk factor (SQ001).
- Thirty-four participants (59%) consider alcohol to be a dangerous risk factor (SQ009).
- Thirty-four participants (59%) consider drugs to be a dangerous risk factor (SQ010).
- Thirty participants (52%) consider speeding to be a dangerous risk factor (SQ011).
- Thirty-four participants (59%) consider not wearing a seat belt to be a dangerous risk factor (SQ012).
- Thirty-six participants (63%) consider running a red light to be a dangerous risk factor (SQ013).
- Thirty-one participants (54%) also consider the passenger to be responsible for preventing an accident (SQ008).
- Twenty-one participants (37%) consider drinking a little alcohol to be harmless (SQ006).
- Fifteen participants (26%) are often told by others to be more careful (SQ003).
- Twenty-two participants (38%) feel safer now because they know more than they did before (SQ001).
- Fifteen participants (26%) see more potential for change in themselves (SQ004).
- Eighteen participants (31%) have a desire to change (SQ005).
- Five participants (8%) would like to try something risky now more than ever (SQ007).

In contrast to the preliminary research, which took place in class during the lesson time, the post-test was given to the participants during the break. This explains the lower participation average.

Through the results of the post-test it could be recognized that the knowledge needs of the participants could be taken up and adequately processed. The application of the training tools was able to trigger the personal development of the participants with regard to a more differentiated way of thinking in relation to their own risk behavior vs. their own safety behavior in road traffic, in order to critically reflect on and review their own behavior.

##### Results risk assessment

The situations depicted on the cards describe risky situations from everyday life, which in some cases result in fines and in many cases have been the cause of traffic accidents in recent years.

The participants were given the opportunity to rate the risk situations on a danger scale between “Dangerous” and “Harmless.” The following graphics show a comparison of the personal assessment of the dangerousness of the situations in relation to the real statistical dangerousness and the classification of the remoteness or closeness to reality on the basis of some exemplary examples.

For the purpose of visualization, the traffic-relevant situational parameters were assigned weighting factors according to their danger levels. Harmless corresponded to weighting factor = 0, medium danger range to weighting factor =1, high-risk to weighting factor =2. The number of cards filed was multiplied by this weighting factor to determine the danger level.

**Figure 5:**
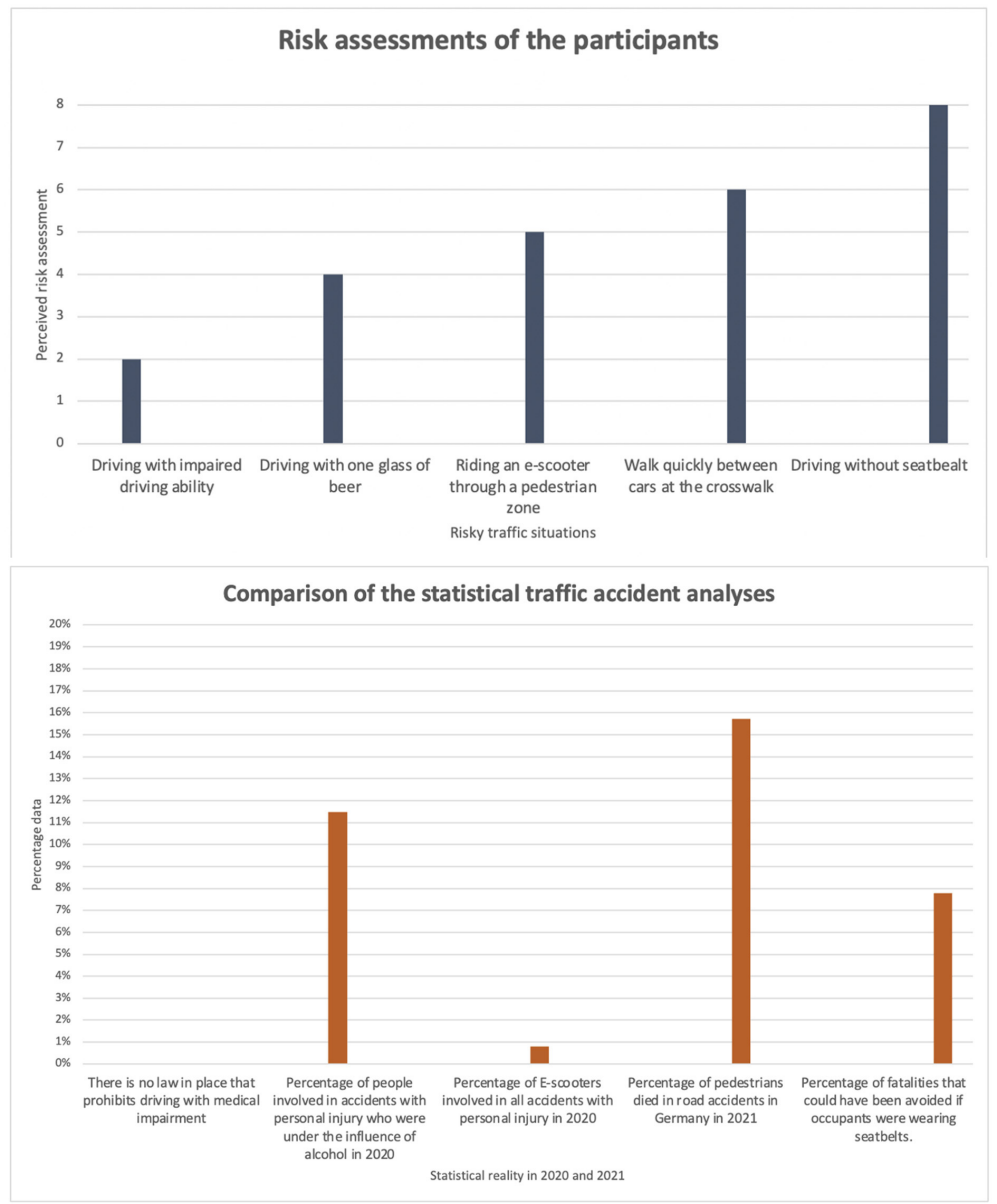
Results of the risk assessment, comparison of personal assessments and statistical reality, **(A)** represents the assessment of the participants, **(B)** represents the actual dangerousness.

In addition, the personally assessed dangerousness is compared with the real statistical dangerousness of the selected situations in the second graph. The overview in Figure 5 shows to what extent the personal estimation of danger corresponds to reality:

It can be seen that the risk of driving without a seat belt and being injured in an accident is rated highest by the participants. In fact, there is a lower risk here compared to the other causes of injury due to the high acceptance of wearing a seat belt in Germany. Compulsory seat belt use was introduced in Germany on January 1, 1976. At first, it applied only to the front seats, then in 1979 it was extended to the back seat. Through ongoing education campaigns, this road safety message seems to have become firmly embedded in the minds of participants.

Participants' assessment of the dangerousness of being able to cross the street as a pedestrian between cars coincides with statistical reality.

Participants perceive the dangerousness of e-scooters to be more risky than the statistical data indicates. One reason for this could be recent news coverage that distorts public perception regarding traffic accidents involving e-scooters (https://www.tagesschau.de/inland/e-scooter-unfaelle-101.html, 09/13/2022).

However, the participants' assessment of their fitness to drive under the influence of alcohol is estimated to be lower than the real statistical accident data shows. The above-mentioned risk of youthfulness could be a reason for this, as participants do not want to admit their own vulnerability in terms of physical reactions after alcohol consumption.

In addition to the level of risk, the situations described also expose dilemmas or raise moral questions. These are suitable for generating discussions on personal norms and values and for initiating changes of perspective.

Significantly striking was that the following risky situations were initially assessed as not dangerous:

- The pedestrian traffic light is just about to change to red, I quickly run across (misjudgement of the traffic rolling up again).
- I bought cabinets at furniture store and load them in the back seat (unsecured load).
- I only had one beer, I can still drive well with that (alcohol influence).
- With fever and headache, I drive quickly to the pharmacy (incorrect assessment of fitness to drive).
- I run across the tracks behind the train (fatal oncoming traffic, so-called “double strike”).
- Riding a bicycle across the pedestrian walkway (risk of collision, pedestrian zone ban).
- I turn right and look very thoroughly and for a long time to the left to see if anyone is coming (traffic coming from the right is not noticed).
- As a pedestrian, I walk quickly between cars at the crosswalk (visual obstruction).
- I pull over on the right-hand side of the freeway because I feel sick (danger on the hard shoulder).
- In a traffic jam I walk across the lane to see when it will go on (danger in the traffic rolling up again) Looking at the cell phone at a red light was only assigned to the medium risk area, although it corresponds to a fine offense according to the fine catalog and can be punishable by points, which corresponds to the assignment of a risky behavior by the legislator and thus administrative injustice (see https://www.bussgeldkatalog.org/tatbestandskatalog-handy/).

**Figure 6:**
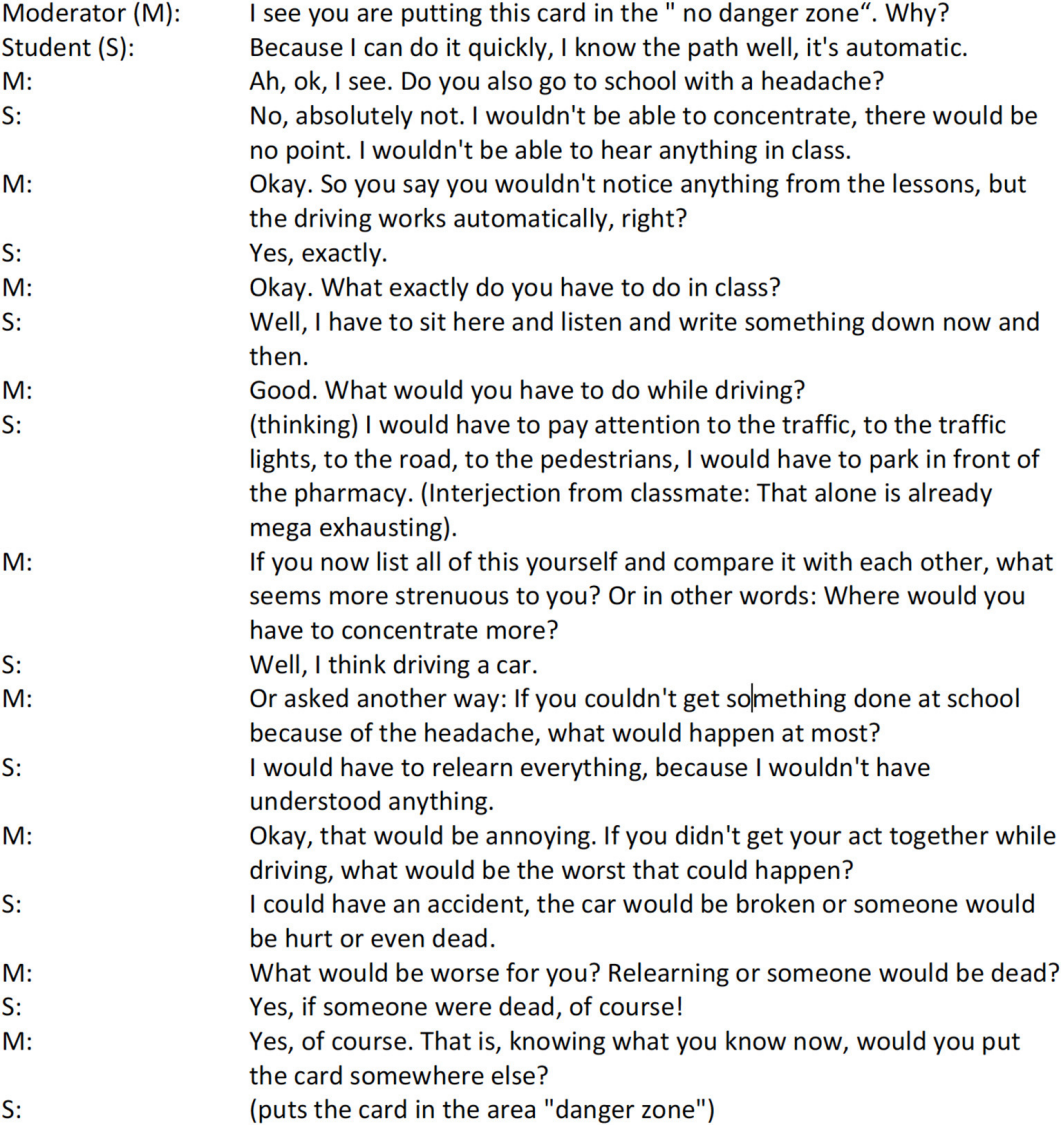
Documentation of a facilitated conversation role card.

An example of clarification of an unrecognized risky situation using Socratic dialog and cognitive restructuring can be found in the following documentation of Figure 6:

At the end of the discussion, no card was in the “Risk” range anymore. According to the post-test evaluation (post-test Appendix), these discussions led the students to new insights.

However, it also became clear that while the importance of the seat belt has received a high level of acceptance, more education is needed in the areas of alcohol, drugs, and general driving skills. In addition, there is still a need for more awareness of the subtle risk situations in bicycle, scooter, and pedestrian traffic.

In the evaluation of the risk scale, it turned out that the division into three risk areas is unfavorable for a scientific analysis. Therefore, in the next implementation, four fields will be taped off on the floor to create a Likert scale and to have the areas “Risk,” “Rather Risk,” “Rather harmless,” and “Harmless” laid out.

Furthermore, it must be noted that a renewed comparison with the personal hazard assessment of the depicted situations on the cards after the discussion is missing. In a future research run, the participants should be asked again after the discussion to check their initially assigned hazard map with regard to the selected filing location and to re-sort it if necessary. This will allow any change in personal assessments of a hazard situation to be measurably tracked.

### Results of qualitative methods

#### Results fatal vision alcohol goggles

By means of field notes and observation protocols, the exercise with the intoxication glasses was researched *via* “Qualitative Observation.” Observation is considered a classic method of qualitative social research. It forms the basis of all empirical research activity (Smart et al., 2013).

The product of the present observation was handwritten notes in class, which were followed by timely comprehensible transcripts on a personal computer.

The observations revealed how the course posed motor and visual difficulties for all participants. These are described in more detail below.

In the course:

- Walking movements deviating to the right or left along an imaginary line.
- Raising the legs when walking.
- Extremely slow walking.
- Extending the arms forward while walking.

When picking up and sorting the cards:

- Targeted grasping of the cards not possible (miss-grasping).
- Often wiping from the side or large area across the floor to grab the card.
- Holding the card alternately near and far in front of the eyes to recognize digit.
- Having to turn card to recognize digit.
- Need to ask group to know digit.
- Cannot place cards parallel.

The recognition as well as the numerical assignment of the digits in the correct order on the cards and a faultless passing of the way was connected with the alcohol-typical failure symptoms.

Initially, witty remarks by the surrounding other participants led to insecurity for the first participants.

Later, participants who had gone through the course became more serious and provided active support from the sidelines.

Serious uncertainty and fright resulted in remarks like, “*Gross, I wouldn't have thought that!”* or “*Oh my God, this is spooky!”*

The participants did not expect the altering effect of alcohol on body reactions and human perception in this form.

This was followed by a detailed discussion of the results, the communication of legal alcohol limits, and possible legal consequences. The effects on private life and the peer group were also included.

In the setting of the exercise “*The Last Two Minute,”* four classmates in the center of the room participated in a simulated car ride.

#### Results of the role play

The role player, who sat in the driver's position, was given a tablet with a pre-installed app. A game with a driving simulation was played on this during the role play. The idea was that the driver should do in the role play exactly what he would do in reality: he should steer a car over a road.

The car simulator on the tablet was a major distraction for both the classmates in the setting and the “bystanders.” They approached the driver and watched him operate the app. The desired driving dynamics and the actual role play of the approaching accident did not occur. Getting into the role situation, “The last two minute before a fatal traffic accident” was unattractive and secondary compared to the game on the tablet. As a result, the author of the article (moderator) changed the moderation and interrupted the tablet car simulator.

In the sense of the design-based research approach, the next iterative step would be to project a car simulator to a larger screen, so that the audience can watch the simulation without approaching the driver.

Now, specific questions to the occupants of the simulation were used to establish the reference to the imminent traffic accident. Options for action, which could positively contribute to preventing such an accident, were to be developed.

Ideas were incorporated into the discussion using the brainstorming method; references to the real world, personal experiences, and hearsay experiences were taken into account. As a result, it was possible to develop the options for action that are suitable for everyday life for the age group.

Finally, it was asked whether the selected methods were suitable for conveying the contents and for bringing about clarification. The degree of suitability was determined by whether the method led to a gain in knowledge. The methods are presented in Table 2.

**Table 2:** Overview of the evaluation of the applied methods, *n* = 57.

**Method**	**Valuation**	**Knowledge gain**
Danger scale with maps	15 participants now see road traffic with different eyes 28 participants got good information about the causes of accidents and risks in road traffic 23 participants found the situations depicted on the cards to be appropriate based on reality 14 participants had no previous knowledge about active participation in road traffic 30 participants did not find the exercise superfluous	Through the survey it could be deduced that the implementation of the event has brought a gain in knowledge for the participants in terms of road safety knowledge
Exercise with the noise goggles	37 participants learned about the effect of intoxication on the human body and the human psyche 32 participants did not find this exercise superfluous	A gain in knowledge about the effect of alcohol and the consequences in the context of participation in road traffic could be achieved
Exercise role play	32 participants said they now know how to be safer and more confident on the road, especially in the context of a peer group dynamic in the car and taking responsibility within a group. 26 participants did not find this exercise superfluous	A gain in knowledge about safety behavior in road traffic could be achieved

## Discussion

### Discussion of the project as a whole

About half of the class, including participants in the “Accompanied Driving from 17” program, participated very lively and attentively in the discussions and contributed their own thoughts. Another quarter of the participants responded well to specific questions, and the last quarter was rather quiet, but seemed to follow cognitively and participated in all the exercises.

This confirms the will to deal with the topics of road safety again in the follow-up to the experienced event. This is also reflected in the results of the post-survey in the actual knowledge gained.

In this first step, a very good overview of the beginning research could be gained. The research results of the impact evaluation, according to which content-related topics from the stage event should be taken up in the follow-up, could be seamlessly incorporated in their demands.

As a result, it is clear that the curricular follow-up of the Crash Course NRW stage event is important because otherwise questions and emotions could remain unanswered, which without clarification could lead to reactance.

This is confirmed by the preliminary survey, in which there is clear evidence of both open questions and emotions that led to reactive responses.

This became clear in the following: on the one hand, only 41 out of 77 participants stated that they would recommend the event to others, as well as 14 participants who answered that they were annoyed by the program. Here, due to the execution of the survey at the time *before* the follow-up with the knowledge about the dynamics of cognitive dissonance, an insecurity and lack of self-efficacy can be assumed. In this respect, the dynamics of the boomerang effect as a result of fear appeals may be considered here as an effect of the stage event.

In the post-test, the statement “*I'm going to try something reckless now”* (Appendix post-test) was used to investigate reactance again. In total, 5 participants indicated here that they would like to do this. This recorded less reactance than was assumed prior to the event. In connection to the result of 22 participants stating after the event that they knew more in terms of road safety than before, it can be concluded that the follow-up and the knowledge imparted here led to a reduction in reactance and the development of behavioral options that provide security. However, it is necessary to verify and confirm this result through further research.

The following overview in Table 3 shows in a comparison to which criteria the follow-up has brought about a change in the participants' ways of thinking and convictions. Furthermore, it should be shown, which needs that the follow-up was able to address and to what extent.

**Table 3:** Comparison of criteria of settings preliminary research (*n* = 77) and post-test (*n* = 57).

**Category**	**Before**	**Afterward**	**Result**
Prior knowledge	14 participants did not know/rather not know that accidents can have these consequences 55 participants found it important to talk about traffic accidents	28 participants stated that they had received good information on the causes of accidents, accident risk behavior, and road safety behavior	Participants could be reached and knowledge could be imparted
Cause of accident	42 participants wanted the causes of the accident to be addressed in the follow-up	28 participants stated that they had received good information on the causes of accidents, accident risk behavior, and road safety behavior.	Participants could be reached and knowledge could be imparted
Prevention	52 participants wished that it was addressed how an accident can be prevented 67 participants wanted to prevent something like this from happening to them	28 participants stated that they had received good information on the causes of accidents, accident risk behavior, and road safety behavior.	Participants could be reached and knowledge could be imparted
Risk situations	51 participants wished that it was addressed which situations in road traffic are dangerous	10 participants did not recognize the danger in the maps shown for various dangerous situations	Some participants could not be reached spontaneously
Laws	46 participants wanted to find out more about the legal situation	28 participants stated that they had received good information on the causes of accidents, accident risk behavior, and road safety behavior.	Participants could be reached and knowledge could be imparted
Health	40 participants wanted to find out more about the influence on health and traffic risks	28 participants stated that they had received good information on the causes of accidents, accident risk behavior, and road safety behavior.	Participants could be reached and knowledge could be imparted
Alcohol, drugs, medication	44 participants wanted to know what exactly causes alcohol, drugs and medication	34 participants consider alcohol and drugs to be a dangerous risk factor, 11 participants consider drinking a little alcohol to be harmless	Most participants could be reached, and some participants could not. Here, reactance could be the cause of persevering in the mind.
Reactant reactions as a result of the fear appeal	1 participant was completely / 5 participants rather afraid to drive a car themselves 14 participants were annoyed 10 participants would have preferred to go outside 7 participants will not / 23 rather not recommend the event	Only 5 participants want to do something reckless now more than ever	No further interviews were conducted thereafter that would have clarified whether follow-up neutralized reactive thinking and strengthened self-efficacy. More research needs to be done here in the future

The latter criterion “Reactant reactions as a result of the fear appeal” confirms the research results of the impact evaluation by Prof. Dr. M. Hackenfort, ZHAW Zurich on the *Crash Course NRW*, according to which reactant behavior was also observed in individual cases.

The focus should be on the participants who developed reactance during the stage event and therefore belong to the risk group. The connection of the content of the stage event to the topics in the schools, then also to the topics of the follow-up, is very important in this context (Hackenfort, 2013, p.155–160).

Here, too, it must be assumed based on this information that reactive behavior in the form of rejection of the event and negation of the danger is hidden behind these statements.

### Quality criteria

When evaluating research results, there is a scientific consensus that research processes must be transparent and comprehensible, and that the quality of the results must be comprehensible in terms of their significance. Quality characteristics of validity, reliability, and objectivity are often used as core criteria in this procedure (Döring and Bortz, 2016, p. 107).

The overview of Table 4 shows the extent to which the quality criteria were taken into account in this pre–post-survey:

**Table 4:** Overview of the quality criteria of the preliminary test and post-test.

**Quality criterion**	**Pretest**	**Post-test**	**Explanation**	**Result**
Objectivity (measurement is independent of the person)	Survey was conducted anonymously by a shared QR code. Instructions for the survey where read to the participants during the introduction	Survey was conducted anonymously by a shared QR code. Instructions for the survey where read to the participants during the introduction	No influence by the interviewer possible	Objectivity is given
Reliability (if the survey is repeated, the measurement result is the same)	Survey was conducted in four classes.	Survey was conducted in four classes.	The results are evenly distributed in all classes. The only significant response in the pre-set is the participant who is afraid to drive a car now.	Survey has a high reliability
Validity (what is to be output is measured)	The reaction to the Crash Course NRW stage event was to be examined in all classes.	The effectiveness of the follow-up to the Crash Course NRW stage event was to be examined in all classes	The questions relate specifically to the two events	Validity is given

With regard to explorative research, an identical transfer of this process in qualitative research hardly finds acceptance. There are two problems with this approach. On the one hand, the systematics as well as the definitions of quantitative quality criteria are not infrequently used blurrily in these transfer attempts; on the other hand, it makes less sense to import criteria for studies that follow a completely different paradigm of scientific theory into the qualitative paradigm (Döring and Bortz, 2016, p. 107). Here, it is more about representativeness in terms of content rather than purely statistical representativeness. More widely accepted than the adoption of quantitative quality criteria is a second approach, which aims to develop its own quality criteria from the logic of qualitative research and to specify the techniques for ensuring them. Against this background, further quality criteria have been developed in qualitative research, which are reviewed in Table 5 below according to Mayring (Godbersen, 2020, p. 11):

**Table 5:** Quality criteria of observational research according to Mayring, *n* = 86.

**Quality criteria**	**Brief description**	**Remarks**
Procedural documentation	Procedural documentation means documenting every step of the analysis. Typically, qualitative content analyses according to Mayring begin with transcriptions. Finally, a results section is written.	Handwritten notes were made during the event, which were compiled into documentation and the production of tables on the PC in the immediate aftermath. These were supported by photographs taken on site.
Argumentative interpretation validation	Interpretations must be justified by argument: Criteria are: (a) adequate prior understanding of the interpretation b) interpretation must be coherent in itself c) alternative interpretations must be sought and verified d) negative interpretation can be an important argument for the validity justification	a) Due to the already existing research by M. Hackenfort on Crash Kurs NRW, it was possible to draw on existing findings and to put the results in the context of already acquired interpretations. b) This approach facilitated the embedding of the new findings into the overall context. The comparison of the existing results with the new results is conclusive and builds logically on each other. c) A comparison with the arguments of fear appeal research in health research (e.g. anti-smoking campaigns) can be made. The results are comparable and reveal the identical psychological dynamics d) The negative effect in fear appeal research, the so-called boomerang effect, which triggers reactance behavior, is a strong measurable factor for the impact of the campaign. This can be seen in negative interpretations
Rule-governed	Quality of interpretation is achieved through a step-by-step, sequential approach. Analysis steps are defined in advance and carried out systematically. These rules concern the material that is included in the analysis and how this is handled.	The content and sequence of the exercises were determined in advance, as they build on each other thematically. The same material was always used for the exercises. The presentation of the respective exercises was always done in the same style. The same materials were used in the same order in all four classes. The content of the discussions varied because the norms, values and attitudes of the students were different.
Proximity to the object	Proximity to the subject means that, at best, interview partners are interviewed in their usual environment. The reason for this is that people always behave somewhat differently in different environments and may also say different things.	During the follow-up training, the participants were in their class within the class group. This is generally a familiar environment. The class teacher, who was also a familiar person, was present throughout. There was a risk that socially desirable behavior would be exhibited in the presence of the class teacher. However, when weighing up the possibilities of coming into contact with pupils of this age for traffic accident prevention campaigns, this represents the best possible variant.
Communicative validation	The validity of the results, the interpretations is thereby checked, by presenting them to the researched and discussing them with them.	The results of the discussions on the exercises were publicly discussed directly in the plenum. Feedback on this could also be given directly below the participants. The results of the anonymous online survey could not be disclosed because they were only evaluated after the school event.
Triangulation	Triangulation can be performed by conducting another qualitative content analysis. Different data sources, different interpreters, different methods or theoretical approaches; results of the different perspectives are compared with each other and formed into a kaleidoscope-like picture composed.	As a result of the outbreak of the pandemic triggered by COVID-19 shortly after the implementation of this follow-up, there was a lockdown and a halt to all school activities. As a result, all *Crash Kurs NRW* events were canceled. No more classes could be taught and therefore no more research results could be collected. Only these four classes could be evaluated against each other.

The impact of the Corona Protection Ordinance allowed only one event to be held, so that only four classes could be compared and the exercises could only be replicated to a limited extent. Whether the lessons learned, as well as the initial consolidations, remain manifest must be tested in follow-up events.

### Answers to the research questions

*Q1: Which didactic methods are suitable to extract and reinforce the safety messages of the stage event?*

*Q2: Which didactic methods are suitable to influence risk behavior in an empathy-expanding way?*

The results of the post-survey (4.2.1) showed that the content-related needs of the participants were met during the follow-up training. The following methods from cognitive behavioral therapy were used:

- Methods that critically examine self-reflective own behavior and attitudes, norms and values, and risk and supposed security (cognitive restructuring according to A. Beck).
- Methods that expose contradictions in thinking (disputation techniques according to A. Beck).
- Methods that contrast possible courses of action and thus offer decision-making options (problem-solving training according to A. Beck).
- Methods that explain safety concepts and provide sufficient information about risks.
- Methods that strengthen self-efficacy (Socratic dialog).

These methods were used together with the tools to make the participants change their thinking. The results related to the effectiveness and usefulness of the tools “*Risk Scale,” “Intoxication Goggles,”* and “*Role Play”* in the *post-test* showed that the methods were successful in initiating a change in thinking. It was possible to launch a new and improved view of risk events in road traffic and to achieve more sensitivity. In addition, improved and new behaviors toward more safety behaviors could be implemented, including a “*Plan B”* to be able to get out of an unsafely driven vehicle *via* pretending to vomit.

Individual needs, such as the need for knowledge about the aftercare of traffic accident victims, as well as the individual emotional situation, such as fear of driving, should at best be taken into account and successfully included in the search process by responding accordingly.

*Q3: How must a follow-up module be designed in terms of content in order to be able to attach important road safety messages in the long term?*

According to the results of the preliminary research (4.1.1), attendants need answers to the following issues after being confronted with real accidents in an accident prevention campaign;

- Accident avoidance strategies.
- Consequences of alcohol and drug use.
- Influence of own health status on road safety.
- Legal knowledge and,
- Accident follow-up.

In the course of the post-survey related to the questions on alcohol, drugs, the learned new safety behavior and related to the own possibilities of change, but also related to the discussions on post-accident care and on physical impairments due to medication on driving ability, it could be determined that these are suitable to establish an adequate exchange on traffic prevention topics and to fill knowledge gaps. The use of the supportive exercises carried out initiates simulations that are close to real life. The exchange of experiences in the peer group during the individual exercises helps to convey the messages adequately. In the post-survey, the respective questions about the exercises reflected the recognizability to everyday life and the usability of the exercises for the transfer into the same.

To solidify these findings, further research should be conducted after the COVID-19 Protective Measures have ended and prevention programs and large audience have resumed in schools.

*Q4: Was reactive behavior generated by the Crash Course NRW stage event and could it be minimized through follow-up?*

The preliminary survey clearly showed that reactive behavior was generated. (Appendix Preliminary Test):

(SQ006) “*I was annoyed” (20%)*.

(SQ007) “*I would have loved to walk out” (13%)*.

(SQ008) “*I have no interest at all in a follow-up” (24%)*.

(SQ008) “*I will not recommend the event to others” (40%)*.

The need to leave the event and the statement not to recommend it to others may be interpreted as rejection and defensiveness toward the event. These responses were not picked up in the post-survey because they could not be acted upon quickly enough.

However, the research at this school has now brought attention to this reactance and another iterative pass will revisit this research question.

The post-test showed reactive behavior even after the follow-up:

(SQ007) “*I'm going to try something reckless now” (9%)*.

In comparison, however, this result is significantly lower than the evidence in the preliminary test. Therefore, it may be concluded that the follow-up training was able to minimize the reactance behavior through the application of the educational and self-efficacy strengthening tools.

### Limitations

#### Limitations of the road safety prevention concept

Following the findings already obtained from the research of Hackenfort et al. on the impact evaluation of the Crash Course NRW stage event, it is not surprising that isolated reactance behavior can be observed despite a well-founded follow-up concept. The confrontation with life-changing events such as accidental injuries or accidental death and also the regulating roles of state legislation are the subject of the lesson follow-up and *per se* suitable to clarify a restriction of personal freedom or also the restriction of personal inviolability. The extent to which there is a willingness to accept one in favor of the other in a weighing of interests depends in particular on the constellations of personal norms and values and cannot always be cognitively restructured within two or three teaching units. Here, a limit of the instructional follow-up must be defined, because the more deeply a normative principle is anchored in personal attitudes, the more time a cognitive reevaluation requires. Also, dynamics such as convenience, group status, or simply dysfunctional habits must be considered here, first detected, and then resolved and transformed. This is often not feasible through a one-time instructional follow-up.

#### Limits of the study

Specific driving behaviors in traffic settings, such as steering, braking, or accelerating behavior, cannot be recorded with this study. Also not covered are personal indicators such as responsiveness, cognitive, or physical parameters that could result in any driving impairment and thus a risk factor for road safety.

### Comparison of results with other studies

Comparable studies that have looked at the effectiveness of promotional campaigns in the road safety sector have also concluded that the effectiveness of an intervention measure increases when it is linked to a traffic education program or sanction (Faus et al., 2021b, p. 21). These findings support the intention to reinforce the safety-related messages from the Crash Course NRW stage event through instructional follow-up and to make them concretely actionable for participants through role simulations that are true to everyday life.

A study from the Dominican Republic that looked at the factor of remembering safety-related messages from traffic prevention campaigns found the following variables to be authoritative: driving a motor vehicle, habitual driving, and possession of a driver's license were causally related to retention of information. Thus, these variables are obviously key elements that distinguish drivers from non-drivers (Faus et al., 2021b, p. 15). However, the instructional follow-up of the Crash Course NRW stage event is aimed not only at young drivers but also at co-drivers aged 16 and above. This therefore includes road users who are not yet drivers and those who are still in training to obtain a driving license. The campaign also intends to sensitize future driving license holders already, to make them aware of the dangers of road traffic and to have a positive influence on them as co-drivers. This was also taken into account in the present research under the aspect of the assumption of responsibility by the co-driver.

### Research gap

In spite of all the attention paid to the appreciative and constructive resolution of inner resistance to a new way of acting and behaving in road traffic, it is important to focus on yet another aspect that has not yet been addressed in this research: the question of whether the presence of police officers in the follow-up training can have a dysfunctional effect. In principle, schools are supposed to carry out the classroom follow-up, but sometimes, schools make use of the support of the police in the form of a traffic safety seminar in order to convey in-depth contents of the Crash Course NRW stage event. Here, the question arises whether the renewed presence of police officers maintains reactance or even exacerbates it. Depending on such a research result, schools and the commissioning ministry could be given a clear recommendation for the implementation of the follow-up training.

## Conclusion

Temporarily interrupted by the COVID-19 pandemic, research should continue with the resumption of *Crash Course NRW* events to produce a valid result.

The obtained results from the post-survey reflect that the applied methods of cognitive restructuring, guided discovery, and structured recognition of potential hazards in the exercises that ultimately led the group to gain knowledge are the right ones (post-survey Appendix).

Important remains basically the message from the fear appeal the trailblazing saving action option to hire. It is important to recognize that effective traffic accident prevention can only be successful when emotionalizing content is applied if stabilizing elements and concrete instructions for action are offered afterward.

The “Get off the gas!” campaign, as a comparable counterpart in traffic accident prevention, also has a very stable body of evidence in this regard (Holte, 2012). In addition to a higher willingness to take risks as a result of sensation seeking, an exaggerated expectation of self-efficacy, i.e., confidence in one's own driving abilities (“I am a particularly good driver”) plays a role (Holte, 2012).

A misjudgment in any direction of one's own ability will always have a negative effect on lived road safety.

We have to accept: Young drivers will always be difficult to convince. Despite all campaigns in favor of attention, this group remains the “problem child” in traffic accident statistics.

Ultimately, it remains to be considered how the contradiction between the search for stimuli and the exaggerated, erroneous confidence in one's own driving skills on the one hand, and the existing expectation of self-efficacy through emotionalizing campaigns on the other, can be resolved. Through the developed training, it can be possible to uncover exactly these contrary erroneous assumptions and shift them into a true reflection of reality, thereby increasing traffic safety behavior.

If making correct decisions relevant to traffic safety is the result, every event has achieved its goal.

## Future research

In a subsequent research, we show how another iterative loop comparatively addresses the issue of fear appeals in an alternating police traffic accident prevention event. Here, we will explore in more depth whether certain criteria repeatedly lead to reactance, which is minimized by consistent intervention:

Effectiveness of intervention measures against the reactance effect in school safety prevention.

Referring to the research findings of Hackenfort et al. a third publication is discussed. It was suggested by Hackenfort et al. to generate further knowledge in an “accident scene analysis” and to transfer it to the knowledge gained in the Crash Course NRW stage event (Klimmt et al., 2015, p. 257). In this way, options for action in traffic safety-relevant behavior should be acquired and thus the boomerang effect minimized.

To achieve this, local accident hotspots can be analyzed and vulnerable danger spots such as school routes can be focused on.

This option can also be used as a follow-up to Crash Course NRW to gain a better understanding of risk situations and safety behavior.

Also building on this study, a further iterative loop of a subsequent publication will focus on such a form of gaining knowledge.

In addition, this form of teaching can target digital teaching in times of lockdown and take into account the current requirements of public health developments:

Traffic space analysis in the blended learning method–a new teaching style of traffic safety in times of COVID-19.

## Data availability statement

The datasets presented in this study can be found in online repositories. The names of the repository/repositories and accession number(s) can be found in the article/Supplementary material.

## Ethics statement

Ethical review and approval was not required for the study on human participants in accordance with the local legislation and institutional requirements. Written informed consent to participate in this study was provided by the participants' legal guardian/next of kin.

## Author contributions

SB developed to the conception and design of the study, wrote the manuscript, conducted the follow-up training and the part taking observation, and developed and evaluated questionnaires for the preliminary research and the post-test. AB and SB led the design of the follow-up training. All authors approved the submitted version.

## Appendix

### Internet sources

Allianz SE. München, Sicherheit im Straßenverkehr (2014). Available online at: https://www.allianz.com/de/presse/news/engagement/gesellschaft/141029-allianz-zur-sicherheit-im-strassenverkehr.html (accessed June 18, 2022).

Alcohol prevention and drug help Cologne. Available online at: https://www.alkoholpraevention-koeln.de/materialien/materialien_rauschbrille_faq.html (accessed June 18, 2022).

Federal Ministry of Transport and Infrastructure: Mobility - Basis for Growth and Employment. Available online at: http://www.bmvi.de/SharedDocs/DE/Artikel/verkehr-und-mobilitaet.html (accessed June 18, 2022).

ICD Code: Systematic index International statistical classification of diseases and related health problems, 10th revision - German Modification. Available online at: https://www.icd-code.de/icd/code/F00-F99.html (accessed June 18, 2022).

Mecklenburg-Western Pomerania state traffic watch: Youth risk. State Traffic Watch Mecklenburg-Western Pomerania. Available online at: https://www.verkehrswacht-mv.de/angebote/junge-fahrer-fahrerinnen (accessed June 18, 2022).

Ministry of the Interior: Applicable decrees, as of 17.12.2020. Decree of the Ministry of the Interior - 41 - 61.02.01 - 3 - dated 19.10.2009

Road safety work of the North Rhine-Westphalia police force. Available online at: https://recht.nrw.de/lmi/owa (accessed June 18, 2022).

Ministry of Education of the State of NRW: Crash Course NRW: Module Handbook. Available online at: https://www.schulministerium.nrw/sites/default/files/documents/Modulhandbuch.pdf (accessed June 18, 2022).

Federal Statistical Office Destatis. Available online at: https://www.destatis.de/DE/Themen/Gesellschaft-Umwelt/Verkehrsunfaelle/_inhalt.html (accessed June 18, 2022). Available online at: https://www.destatis.de/DE/Themen/Gesellschaft-Umwelt/Verkehrsunfaelle/Publikationen/Downloads-Verkehrsunfaelle/unfaelle-18-bis-24-jaehrigen-5462406197004.pdf?__blob=publicationFile (accessed June 18, 2022).

Catalog of offenses: Cell phone use. Available online at: https://www.bussgeldkatalog.org/tatbestandskatalog-handy/ (accessed June 18, 2022).

University of Cologne, Institute for Physics Didactics: Crash Course NRW: Experience Reality - Really Hard. Available online at: https://crashkurs-nrw.uni-koeln.de/ (accessed June 18, 2022).

University of Cologne, Institute for Physics Didactics: Crash Course NRW: Practice acting in role play. Available online at: https://crashkurs-nrw.uni-koeln.de/handeln-ueben (accessed June 18, 2022).

Tagesschau: Mehr als 2100 E-Scooter Unfälle. Available online at: https://www.tagesschau.de/inland/e-scooter-unfaelle-101.html (accessed September 13, 2022).

Traffic Accident Victim Assistance Germany e.V. (VOD). Available online at: https://vod-ev.org/vorstand/ (accessed June 18, 2022).
